# New Appliances and Things Medical

**Published:** 1897-08-14

**Authors:** 


### NEW APPLIANCES AND THINGS MEDICAL.

[We shall be glad to receive, at our Office, 28 & 29, Southampton Street, Strand, London, W.C., from the manufacturers, specimens of all new preparations and appliances which may be brought out from time to time.]

### A FLOOR SCRUBBING MACHINE.

A machine which marks a new departure in lifting the burden of floor scrubbing from human muscles and putting it into a mechanical contrivance has recently been invented, and is now to be seen at 81, Cannon Street. Valuable as is the work that can now be done by its aid, the developments and adaptations which may possibly in time make it a domestic necessity are still more important. The machine is much like a lawn-mower in size, and the principle upon which it is constructed is not dissimilar. The rotatory knives which cut the grass are replaced by two rotatory brushes which scrub the floor. A tank containing boiling water, soap, and a disinfectant, if wished, is placed immediately over these brushes and the lye is evenly supplied through a perforated pipe as we see in a cart for watering the streets. This is the cleansing apparatus. The drying apparatus is entirely novel. A round woollen cloth is arranged over rollers and a brush and passes between a wringer, which extracts and consigns the dirty water to a second tank. The cloth is pressed evenly on the wet surface of the floor by means of the third roller brush; it is then carried by means of rollers to the wringer, and thence over the brush once more, so that in use the cloth is continuously passing over the damp floor and the moisture in process of being extracted by the wringer. The largest machine is twelve inches wide, and cleanses a strip of flooring that width when passed over it. The price at present runs at £1 an inch. We hear that it is the intention of the owners to make the price as moderate as possible, as they are certain the demand will be sufficiently large to enable them to count on quick returns. There are two drawbacks, and it is difficult to see how they could be avoided. The one is the weight (it would only be possible to employ women labour on the smaller machine as it is quite as heavy as a lawn-mower); the other is that the operator must perforce walk on the newly-scrubbed floor. In the latter case, perhaps, the difficulty might be obviated by the user wearing felt-soled shoes. Hospital authorities, shopkeepers, and restaurant owners and others who have to see to the cleansing of large areas cannot fail to find these machines of great service and economy, whilst housekeepers will await with interest their adaptation to the requirements of smaller *ménages*.

### OLD BRANDY. (GAUTIER FRÈRES.)

(Brown, Gore, and Co., 23, Great Tower Street, E.C.)

The Cognac brandy districts have been of late years so devastated by the phylloxera that it is frequently difficult to obtain brandies which have been sufficiently matured by age so as to fit them for medicinal purposes. Messrs. Gautier Frères, of Cognac, have submitted to us samples of liqueur brandy which is claimed to be 20 years old. On analysis it gave results showing it to be of good alcoholic strength, 18·6 degrees under proof, and not too highly liqueured, the extractives being 1·25 per cent. and acidity ·058 per cent. In flavour and bouquet it has all the characters of brandy which has been matured by long storage. It is a brandy highly suitable for general use or for medicinal purposes when a liqueur brandy is desirable.

